# Effect of Atorvastatin and Pioglitazone on Plasma Levels of Adhesion Molecules in Non-Diabetic Patients With Hypertension or Stable Angina or Both

**DOI:** 10.14740/jocmr2178e

**Published:** 2015-06-09

**Authors:** Vishwanath Pattan, Sandeep Seth, Waqas Jehangir, Balram Bhargava, Subir Kumar Maulik

**Affiliations:** a Department of Pharmacology, All India Institute of Medical Sciences, New Delhi, India; b Department of Cardiology, All India Institute of Medical Sciences, New Delhi, India; c Raritan Bay Medical Center, Perth Amboy, NJ 08861, USA

**Keywords:** hsCRP, sICAM-1, sVCAM-1, Atorvastatin, Pioglitazone

## Abstract

**Background:**

It was to study the effect of atorvastatin, pioglitazone and their combination on plasma levels of adhesion molecules in patients with hypertension or stable angina or both.

**Methods:**

It was an open-label, randomized parallel-group study. Forty-five atorvastatin-naive patients with hypertension or stable angina or both, were randomized to receive either atorvastatin (19 patients; 10 mg OD for 12 weeks) or pioglitazone (26 patients; 30 mg OD for 12 weeks). Another group of 30 patients who were already on atorvastatin were put on add-on pioglitazone therapy (pioglitazone (15 mg OD) + atorvastatin (10 mg OD) for 12 weeks). Plasma high-sensitivity C-reactive protein (hsCRP), soluble intercellular adhesion molecule-1 (sICAM-1) and soluble vascular cell adhesion molecule-1 (sVCAM-1) levels were measured at baseline and after 12 weeks of therapy.

**Results:**

Atorvastatin monotherapy significantly reduced plasma sICAM-1, but pioglitazone monotherapy did not produce any significant effect. Addition of pioglitazone in patients already receiving atorvastatin also significantly reduced plasma sICAM-1 level. However, there was no significant change in plasma hsCRP and sVCAM-1 levels in any of the groups after 12 weeks of therapy.

**Conclusion:**

There is therapeutic advantage of combining pioglitazone and atorvastatin on plasma sICAM-1 levels.

## Introduction

Adhesion molecules like soluble intercellular adhesion molecule-1 (sICAM-1) and soluble vascular cell adhesion molecule-1 (sVCAM-1) have been shown to be raised in early stages of atherosclerosis [1] and mediate firm attachment of monocytes and T cells to the endothelium [2], and favor their transmigration into the vascular intima [3]. The entrapped monocytes in the intima differentiate into macrophages and form foam cells which are critical in initiation and progression of atherosclerotic plaques. Adhesion molecules have been identified as the potential biomarkers to predict future cardiovascular events [4-6]. In view of this fact, there have been increasing efforts to direct pharmacotherapy to reduce the plasma levels of adhesion molecules and several other surrogate markers that reflect endothelial dysfunction and future cardiovascular events [7, 8].

In addition to decreasing cholesterol synthesis, statins have pleiotropic effects that include improvement of endothelial dysfunction, increased nitric oxide bioavailability, antioxidant properties, inhibition of inflammatory responses and stabilization of atherosclerotic plaques [9, 10]. Many of these pleiotropic effects are attributed to inhibition of synthesis of isoprenoid units farnesyl pyrophosphate (FPP) and geranylgeranyl pyrophosphate (GGPP), which serve to regulate the functions of monomeric GTPases like Ras and Rho family of proteins [11, 12]. They have many favorable effects on endothelial dysfunction and future cardiovascular events [7-9]. Pioglitazone, a thiazolidinedione, is a peroxisome proliferator-activated receptor gamma (PPAR-γ) agonist which in addition to its insulin sensitizing action, is also reported to have many favorable effects on endothelial dysfunction (including antiatherogenic properties [13, 14]) and future cardiovascular events in both diabetic and non-diabetic patients [15, 16]. PPAR-γ regulates the recruitment of monocytes to endothelial cells [17], inflammatory responses in macrophages [18], and the proliferation and migration of vascular smooth muscle cells [19]. Few studies have also reported the beneficial effects of pioglitazone on adhesion molecules in diabetic patients [20, 21]. But hitherto, there have been no studies to report the effect of pioglitazone on adhesion molecules in non-diabetic patients.

Since atorvastatin and pioglitazone exert their effects at different molecular levels that control expression of adhesion molecules, we hypothesized that pioglitazone might have synergistic effect in combination with atorvastatin on reduction of adhesion molecules.

## Methodology

The present study was conducted according to the principles expressed in the Declaration of Helsinki and with Institutional Ethics Committee’s approval (No. A-29/29.1.2007). All patients gave informed written consent to participate before the start of the study. It was an open-label randomized parallel-group study.

### Study population

Patients with hypertension (stage I, systolic BP < 160, diastolic BP < 100; JNC7) [22] or stable angina or both between 35 and 65 years belonging to both sexes who were diagnosed based on history, clinical examination, electrocardiogram (ECG) or angiography, were recruited from outpatient clinic.

Patients with type 2 diabetes mellitus (fasting plasma glucose (FPG) > 125 mg/dL), hepatic/renal dysfunction, tobacco consumption in any form within previous 6 months, pregnant or lactating women, congestive heart failure (CHF) (New York Heart Association (NYHA) class II to IV), recent myocardial infarction (within 6 weeks), history of unstable angina, history of allergy to statins or pioglitazone, severe or uncontrolled hypertension (stage 2 hypertension, > 160/100 mm Hg by JNC7 [22]) were excluded from the study.

### Study protocol

A total of 200 outpatients with hypertension or stable angina were screened at Department of Cardiology, All India Institute of Medical Sciences (AIIMS), New Delhi from March 2007 to November 2008, of which 75 non-diabetics were found eligible. They were stratified into: 1) those already on atorvastatin; and 2) atorvastatin-naive patients. Out of these, 45 atorvastatin-naive patients were randomized to receive either atorvastatin (group 1, 10 mg OD for 3 months; n = 19) or pioglitazone (group 2, 30 mg OD for 3 months; n = 26). Computer-generated random number sequence was obtained and the sealed envelope method was used for randomization [20] of atorvastatin-naive patients. Similarly another group of 30 patients who were already taking atorvastatin (for 4 - 6 months) were assigned to receive add-on pioglitazone therapy (group 3, pioglitazone (15 mg OD) + atorvastatin (10 mg OD) for 3 months; n = 30). Since statins have a mortality benefit in ischemic heart disease, most patients with stable angina were already on atorvastatin and hence were put on add-on pioglitazone therapy. Plasma concentrations of sICAM-1, sVCAM-1 and hsCRP, were measured at baseline and 12 weeks of treatment. The patients were assessed for liver enzymes (aspartate aminotransferase (AST) and alanine aminotransferase (ALT)), and renal function parameters (blood urea nitrogen (BUN), blood urea and serum creatinine) at baseline and after 12 weeks. Patients were educated about the adverse effects and informed to report immediately if they had any symptoms suggestive of hypoglycemia, and adverse effects suggestive of myopathy or hepatic dysfunction. Weekly telephonic interview was also carried out to elicit history suggestive of these adverse effects.

### Measurements of hsCRP, sICAM-1 and sVCAM-1

After overnight fasting, 3 mL of venous blood sample was drawn into the heparinized poly-vinyl tube. The samples were centrifuged within 30 min at 1,000 g for 10 min at room temperature. Plasma samples were pipetted into 1.5 mL eppendorf tubes and stored at -70 °C until analysis. The hsCRP (Calbiotect Inc., USA), sICAM-1 (Diaclone, France) and sVCAM-1 (Diaclone, France) were estimated by a person blinded to the study groups. Plasma samples were thawed once at room temperature.

### Statistical analysis

The baseline characteristics of patients were compared using analysis of variance (ANOVA). The effect of treatment in each group was assessed using Wilcoxon signed-rank test. The differences between the outcomes of the three study groups were compared using Kruskal-Wallis test. The P-value of < 0.05 was considered statistically significant. Seventy-five patients were enrolled in the study, of which 59 patients completed the follow-up after 12 weeks and 16 patients (five in group 1, six in group 2 and five in group 3) were lost to follow-up. Parameters with skewed distribution (hsCRP, sICAM-1 and sVCAM-1) were also analyzed after log transformation to normalize their distribution. Effect of treatment in each group was assessed using paired *t*-test and the differences between the outcomes of the three study groups were compared using ANOVA.

## Results

The normally distributed observations (like blood pressure, fasting blood glucose, AST, and ALT) are expressed as mean ± SD. The skewed observations of hsCRP, sICAM-1 and sVCAM-1 are expressed in median and inter-quartile range (IQR).

The study cohort consisted of comparable baseline characteristics (Table 1) with regard to variables of age, blood pressure, fasting plasma glucose, lipid profile, liver and renal function parameters. There was male preponderance in all the study groups. The proportion of patients with stable angina was higher in group 3 (64%) when compared to group 1 (21.4%) and group 2 (40%).

**Table 1:** Baseline Characteristics

Variable	Group 1	Group 2	Group 3
Number of patients	14	20	25
Sex (M/F)	10/4	14/6	19/6
Age (years)	51 ± 7.9	50.3 ± 10.0	49.8 ± 9.1
FPG (mg/dL)	89.2 ± 10.6	91.2 ± 10.2	91.9 ± 11.2
SBP (mm Hg)	135.8 ± 7.1	131.8 ± 10.2	130.8 ± 10.9
DBP (mm Hg)	87.7 ± 5.9	86.3 ± 4.6	82.9 ± 5.4
Serum AST (IU/L)	36.2 ± 4.6	35.9 ± 4.2	36.7 ± 4.8
Serum ALT (IU/L)	35.7 ± 4.8	34.8 ± 3.7	35.2 ± 4.3
Blood urea (mg/dL)	29.7 ± 6.5	29 ± 6.4	29.1 ± 6.3
Serum creatinine (mg/dL)	1.07 ± 0.20	1.1 ± 0.2	1.0 ± 0.2
LDL cholesterol	105 ± 28	110 ± 32	98 ± 24
Triglycerides	144 ± 110	152 ± 96	140 ± 100
Hypertension without stable angina	11	12	9
Stable angina without hypertension	1	5	12
Hypertension and stable angina	2	3	4

Variables expressed in mean ± SD. SBP: systolic blood pressure; DBP: diastolic blood pressure; FPG: fasting plasma glucose; AST: aspartate aminotransferase; ALT: alanine aminotransferase.

The proportions of patients with isolated hypertension were higher in group 1 (78.6%) and group 2 (60%) when compared to group 3 (36%).

There were no statistically significant differences in the baseline levels of hsCRP (P = 0.09), sICAM-1 (P = 0.65) and sVCAM-1 (P = 0.12) (Table 2) in all the study groups which were in intermediate range (1 - 3 mg/L).

**Table 2:** Effect of Treatment on Adhesion Molecules Plasma Values of hsCRP, sICAM-1 and sVCAM-1 at Baseline and 12-Week Follow-Up

	Group 1 (n = 14)		Group 2 (n = 20)		Group 3 (n = 25)	P^a^
Baseline						
hsCRP	0.63 (0.01 - 3.42; 2.07)		1.91 (0.01 - 5.04; 2.41)		2.54 (0.18 - 5.05; 2.25)	0.09
sICAM-1	357.4 (48.0 - 700.9; 310.4)		401.7 (182.1 - 751.1; 229.8)		435.3 (148.8 - 899.6; 240.5)	0.65
sVCAM-1	314.5 (129.0 - 858.9; 362.9)		361.0 (96.7 C 1,142.5; 466.8)		536.3 (76.6 - 588.7; 745.7)	0.12
12-week follow-up		P^b^		P^b^		P^b^
hsCRP	0.91 (0.01 - 8.24; 2.54)	0.72	1.01 (0.01 - 4.03; 4.79)	0.91	3.41 (1.03 - 8.68; 3.45)	0.32
sICAM-1	189.2 (3.2 - 531.5; 265.6)	0.01	396.5 (59.7 - 935.6; 363.1)	0.23	249.5 (2.5 - 789.9; 339.7)	0.004
sVCAM-1	188.9 (0.1 - 2,989.5; 1,209.5)	0.84	576.6 (0.1 - 1,294.4; 984.8)	0.35	439.5 (0.1 - 2,632.0; 1,790.3)	0.44

Values are expressed as median (min-max; inter-quartile range). P^a^: P-value when base line plasma values of hsCRP, sICAM-1 and sVCAM-1 are compared between the study groups. P^b^: P-value when post treatment plasma values of hsCRP, sICAM-1 and sVCAM-1 are compared to their respective baseline values in each treatment group. P-value < 0.05 was considered statistically significant.

### Effect of treatment on adhesion molecules (sICAM-1 and sVCAM-1) and hsCRP

**Figure 1:**
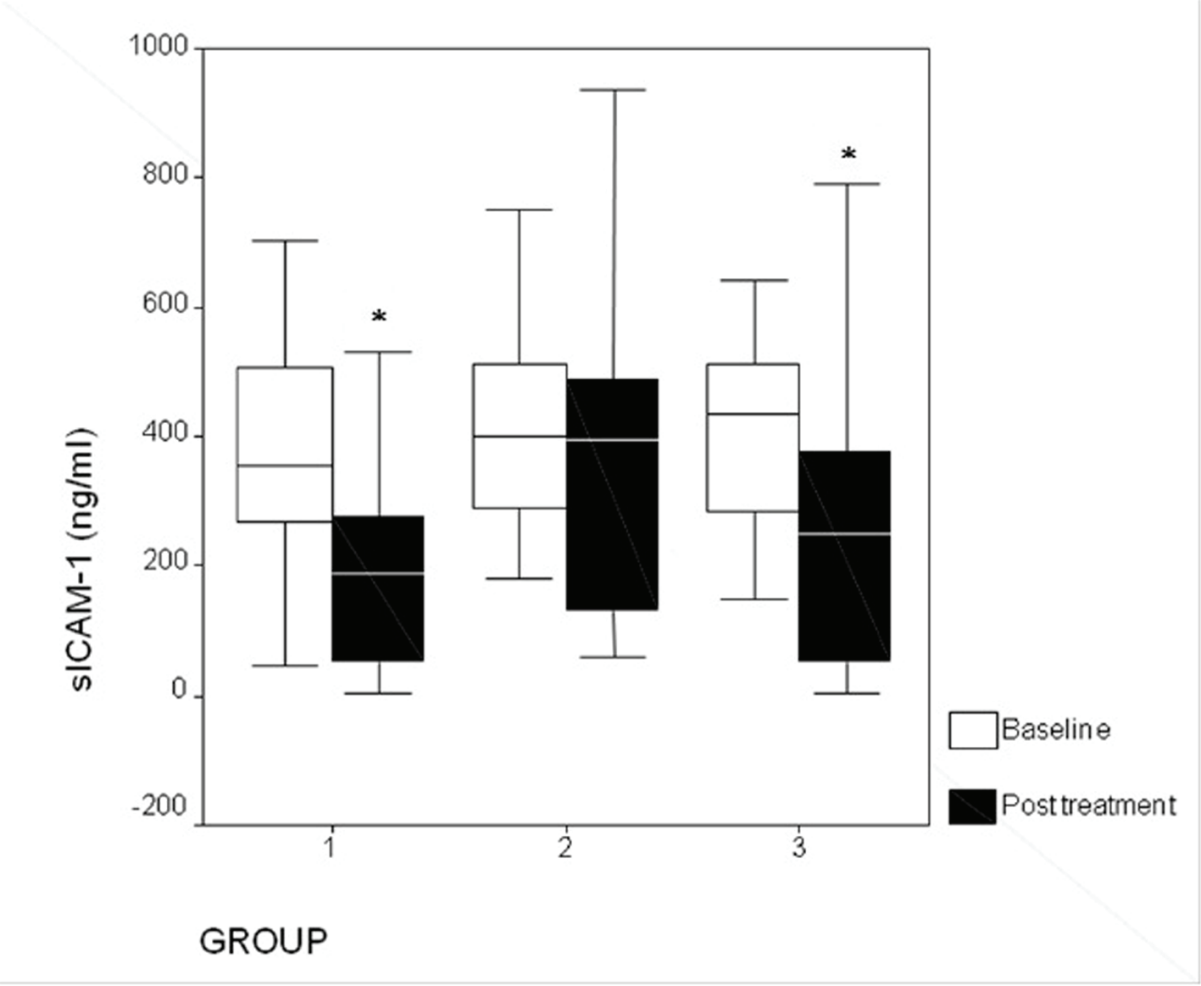
Changes in plasma sICAM-1 levels after treatment (*P < 0.05). Group 1: atorvastatin; group 2: pioglitazone; group 3: atorvastatin + pioglitazone.

**Figure 2:**
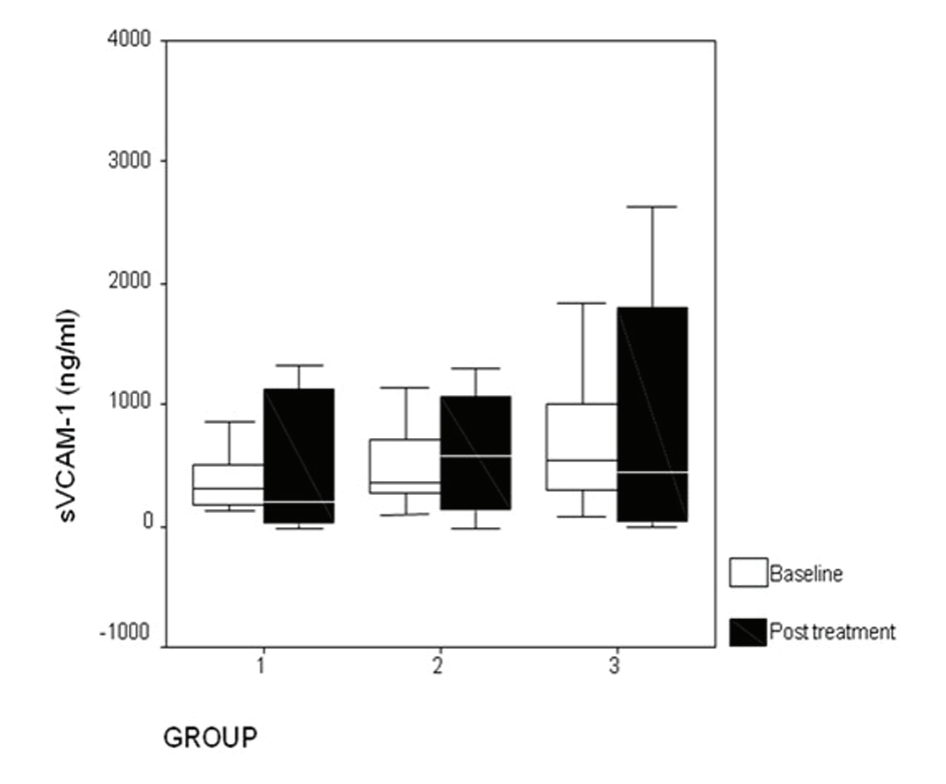
Changes in plasma sVCAM-1 levels after treatment. Group 1: atorvastatin; group 2: pioglitazone; group 3: atorvastatin + pioglitazone.

**Figure 3:**
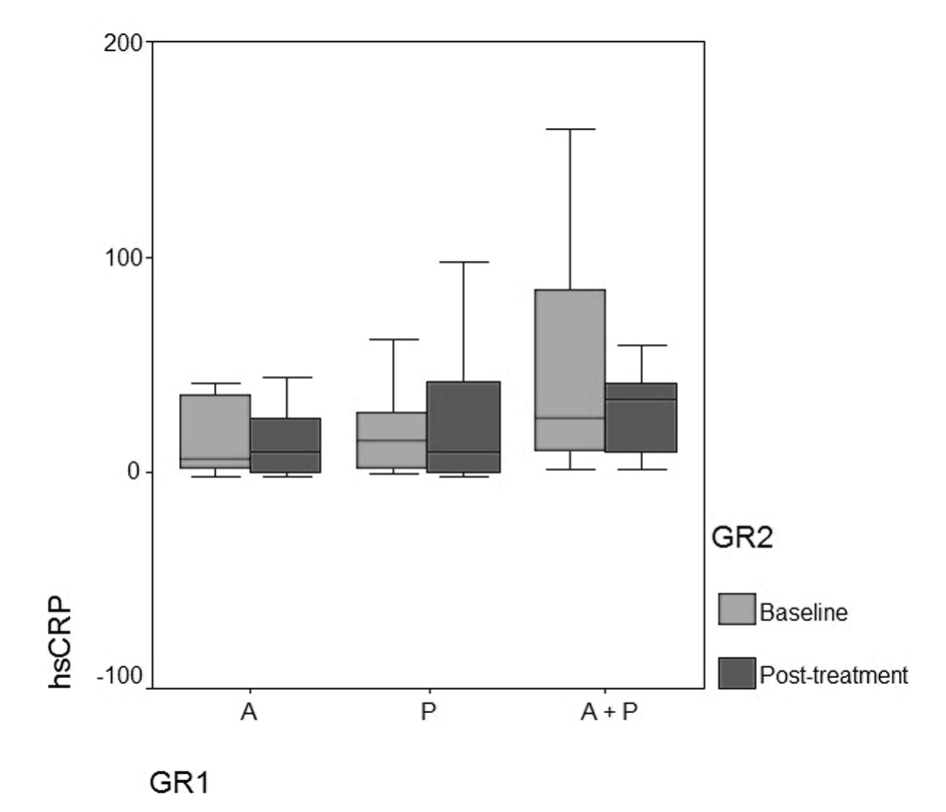
Changes in hsCRP levels after treatment. A: atorvastatin; P: pioglitazone; A + P: atorvastatin + pioglitazone.

Addition of pioglitazone (15 mg/day) to patients already taking atorvastatin further reduced plasma levels of sICAM-1 (P = 0.004) (Fig. 1) from baseline. Atorvastatin monotherapy produced significant (P = 0.01) reduction in plasma levels of sICAM-1, but this was not seen not with pioglitazone monotherapy (P = 0.23). There was no significant (P > 0.05) difference in plasma sICAM-1 between patients receiving atorvastatin and patients receiving combination of atorvastatin and pioglitazone. When compared to baseline, there was no significant change (P > 0.05) in levels of sVCAM-1 (Fig. 2) and hsCRP (Fig. 3) levels in any of the study groups after 12 weeks of therapy (Table 3).

**Table 3:** Liver and Renal Function Parameters at Baseline and 12-Week Follow-Up

Variable	Group 1			Group 2			Group 3		
	Baseline	3 months	SS	Baseline	3 months	SS	Baseline	3 months	SS
Serum AST (IU/L)	36.2 ± 4.6	36.7 ± 4.9	NS	35.9 ± 4.2	36.0 ± 4.4	NS	36.71 ± 4.8	37.0 ± 4.9	NS
Serum ALT (IU/L)	35.7 ± 4.8	36.0 ± 4.6	NS	34.8 ± 3.7	35.1 ± 4.2	NS	35.2 ± 4.3	36.0 ± 4.2	NS
Blood urea (mg/dL)	29.7 ± 6.5	29.9 ± 6.8	NS	29.0 ± 6.4	29.3 ± 6.9	NS	29.1 ± 6.3	29.5 ± 6.6	NS
Serum creatinine (mg/dL)	1.0 ± 0.2	1.1 ± 0.3	NS	1.1 ± 0.2	1.1 ± 0.2	NS	1.0 ± 0.2	1.1 ± 0.2	NS

SS: statistical significance; NS: not significant (P > 0.05). Values are expresses as mean ± SD.

Liver (serum AST and ALT) and renal functions (blood urea and serum creatinine) were within the normal range during the study period in all the three groups.

## Discussion

In the present study, we evaluated the effect of adding pioglitazone to patients already receiving atorvastatin on plasma levels of adhesion molecules (sICAM-1 and sVCAM-1), for any synergistic effect. Addition of pioglitazone (15 mg/day), a PPAR-γ agonist, significantly reduced plasma levels of sICAM-1 in patients already receiving atorvastatin. Atorvastatin monotherapy also produced significant reduction in plasma levels of sICAM-1.

Addition of pioglitazone in atorvastatin-treated patients produced similar improvement in plasma sICAM-1 levels as that of atorvastatin monotherapy in atorvastatin-naive patients. This indicates that addition of pioglitazone was able to benefit sICAM-1 levels in patients despite being already treated with atorvastatin for 4 - 6 months, which was similar to the benefit obtained from 12-week atorvastatin monotherapy in atorvastatin-naive patients. The baseline levels of sICAM-1 were higher in patients receiving add-on pioglitazone despite being on atorvastatin treatment for 4 - 6 months. This might be explained by the higher proportion of stable angina patients with higher baseline sICAM-1 levels in this group (owing to progressive atherosclerosis with plaque changes), and atorvastatin alone for 4 - 6 months was not sufficient to bring the sICAM-1 to lower levels as that obtained by group receiving atorvastatin monotherapy, who had lesser proportion of stable angina patients and lower baseline sICAM-1 levels.

This suggests that pioglitazone may further reduce plasma sICAM-1 levels by a synergistic effect with atorvastatin in patients whose sICAM-1 levels are high at baseline.

An interesting finding in our study was that although pioglitazone was effective in reducing plasma sICAM-1 level at 15 mg/day dose when added to patients already receiving atorvastatin, pioglitazone monotherapy at higher dose of 30 mg/day did not have significant effect on plasma sICAM-1 levels. Although the mechanism of this effect is not clear, it is possible that pioglitazone reduces plasma sICAM-1 levels in non-diabetic patients only in the presence of atorvastatin. Though there are no studies showing effect of pioglitazone on adhesion molecules in a non-diabetic cohort, studies have shown contradictory evidences regarding effect of PPAR-γ agonists on adhesion molecules in type 2 diabetic patients. Similar result was obtained by Marx and colleagues (2003) who observed no statistically significant change in levels of sICAM-1 and sVCAM-1 after 12 weeks of treatment with another PPAR-γ agonist rosiglitazone in diabetic patients [23]. However, Takase et al (2007) observed significant reduction in sICAM-1 and sVCAM-1 after 1 month of pioglitazone treatment in a type 2 diabetic cohort [20].

There was no significant change in plasma sVCAM-1 levels after 12-week treatment in any of the study groups. Although there is debate as to which amongst the adhesion molecules is a better marker of subsequent cardiovascular events, there is an accumulating evidence that sICAM-1 is a potential biomarker which is raised in early stages of atherosclerosis [1, 24] and predicts future cardiovascular events both in asymptomatic healthy patients [5, 25] and patients with coronary heart disease [26]. On the other hand, sVCAM-1 has failed to show such consistent association in studies [5, 26]. This indicates that sICAM-1 may serve as a better marker of subsequent cardiovascular events than sVCAM-1 which may predict the benefit of the pharmacological intervention on cardiovascular outcome. However, there are studies that refute this evidence [6, 27].

There are several studies which support the benefit of use of pioglitazone in non-diabetic patients with CAD [15, 16, 28] and hypertension [29, 30]. Pioglitazone has been reported to exert favorable effects in both diabetic and non-diabetic patients, independent of its effect on glucose metabolism [16, 28]. Since adhesion molecules are predictive of increased risk for subsequent cardiovascular events [4-6], the effect of pioglitazone on adhesion molecules in non-diabetic patients might have therapeutic benefits. This is the first study which reports a favorable effect of combining pioglitazone with atorvastatin on adhesion molecules sICAM-1 in non-diabetic patients.

In our study, there was no statistically significant change in hsCRP levels in any of the study groups. Although initially considered to be a promising biomarker for cardiovascular risk stratification, recently hsCRP levels are reported to be strongly related to many other potential confounding factors that influence CHD incidence and mortality [31]. Nissen and colleagues observed that treatment with 40 mg pravastatin (about 10 mg atorvastatin) for 18 months did not produce significant reduction in hsCRP as compared to significant reduction in hsCRP obtained by treatment with 80 mg atorvastatin for 18 months [32]. This indicates that high-dose atorvastatin therapy may benefit hsCRP levels by overriding the confounding influences affecting hsCRP. But this benefit may not be seen at lower doses. Similar to our study result, no significant change in hsCRP was seen in non-diabetic patients after 3 months of treatment with pioglitazone by Staniloae et al (2007) [28]. Hanefeld and colleagues reported significant reduction in hsCRP levels in non-diabetic patients after 12 weeks of treatment with pioglitazone [33]. However, the latter study involved patients with elevated hsCRP levels (> 3 mg/L) at baseline. This indicates that benefit of pioglitazone in non-diabetic patients may be clinically evident only when baseline hsCRP levels are high.

In the present study, patients in all the study groups were using aspirin [34], angiotensin converting enzyme (ACE) inhibitors [35] and calcium channel blockers [36], which have been reported to play a role in reducing the plasma levels of adhesion molecules. However, since these drugs were used by patients with hypertension (calcium channel blockers, ACE inhibitors) and stable angina (aspirin, ACE inhibitors) in all the study groups, their effect may be equally distributed.

### Study limitations

The sample size of the study was small. It was an open-label study. The distribution of stable angina patients was not balanced among the three study arms. To better understand, whether the combination of atorvastatin and pioglitazone would be synergistic in reducing plasma levels of sICAM-1, the combination therapy of atorvastatin and pioglitazone should be started in patients who are not already receiving atorvastatin instead of add-on pioglitazone therapy, ensuring equal distribution of the hypertensive and stable angina patients between groups.

### Conclusion

Atorvastatin monotherapy reduces plasma levels of sICAM-1 and might have therapeutic potential in cardiovascular risk reduction. Addition of pioglitazone reduces plasma levels of sICAM-1 in patients already receiving atorvastatin, and may provide additional benefit in the reduction of subsequent cardiovascular events.
